# Availability of adequately iodized salt at the household level in Ethiopia: A systematic review and meta-analysis

**DOI:** 10.1371/journal.pone.0247106

**Published:** 2021-02-16

**Authors:** Alehegn Aderaw Alamneh, Cheru Tesema Leshargie, Melaku Desta, Molla Yigzaw Birhanu, Moges Agazhe Assemie, Habtamu Temesgen Denekew, Yoseph Merkeb Alamneh, Daniel Bekele Ketema

**Affiliations:** 1 Department of Human Nutrition and Food Sciences, College of Health Sciences, Debre Markos University, Debre Markos, Ethiopia; 2 Department of Public Health, College of Health Sciences, Debre Markos University, Debre Markos, Ethiopia; 3 Faculty of Health, University of Technology Sydney, Sydney, Australia; 4 Department of Midwifery, College of Health Sciences, Debre Markos University, Debre Markos, Ethiopia; 5 College of Health Sciences, Debre Markos University, Debre Markos, Ethiopia; 6 Departments of Biomedical Sciences, Schools of Medicine, Debre Markos University, Debre Markos, Ethiopia; Monash University Malaysia, MALAYSIA

## Abstract

**Background:**

Iodine deficiency disorder (IDD) is a global, regional, and national public health problem that is preventable. Universal salt iodization is a worldwide accepted strategy to prevent IDD. The level of iodine in the salt should be adequate at the household level (≥15ppm). Though there was fragmented evidence on the proportion of adequately iodized salt at the household level in Ethiopia, the national level proportion of adequately iodized salt at the household level was remaining unknown. Therefore, this systematic review and meta-analysis estimated the pooled proportion of adequately iodized salt at the household level in Ethiopia from 2013–2020.

**Method:**

We systematically searched the databases: PubMed/MEDLINE, Google Scholar, and Science Direct for studies conducted in Ethiopia on the availability of adequately iodized salt at the household level since 2013. We have included observational studies, which were published between January first, 2013, and 10 August 2020. The report was compiled according to the Preferred Reporting Items for Systematic Reviews and Meta-Analysis (PRISMA) guidelines. The quality of included studies was scored based on the Newcastle Ottawa quality assessment scale adapted for cross-sectional studies. The data were extracted in Microsoft excel and analyzed using Stata version 14.1 software. We employed a random-effects model to estimate the pooled proportion of adequately iodized salt at the household level in Ethiopia. The presence of statistical heterogeneity within the included studies was evaluated using the I-squared statistic. We used Egger’s regression test to identify evidence of publication bias. The pooled proportion with a 95% confidence interval (CI) was presented using tables and forest plots.

**Results:**

We screened a total of 195 articles. Of these, 28 studies (with 15561 households) were included in the final systematic review and meta-analysis. In Ethiopia, the pooled proportion of adequately iodized salt at the household level was 37% (95% CI: 28, 46%). The subgroup analyses of 28 studies by residence revealed that the pooled proportion of adequately iodized salt at the household level was 32% (95% CI: 29, 35%) and 48% (95% CI: 31, 66%) in rural and urban areas, respectively. Based on geographic location, the highest proportion was found in Addis Ababa (81%; 95%CI: 78, 83), and the lowest proportion found in Dire Dawa (20%; 95%CI: 17, 22). Besides, the proportion of adequately iodized salt at the household level was significantly increased during 2017–2020 (42%; 95% CI: 30, 53%) as compared with 2013–2016 (27%; 95% CI: 17, 39%).

**Conclusions:**

In Ethiopia, the pooled proportion of adequately iodized salt at the household level was very low as compared to the world health organization’s recommendation. Thus, the Federal Ministry of Health of Ethiopia and different stakeholders should give more attention to improve the proportion of adequately iodized salt at the household level.

## Background

Iodine is a chemical element that is essential for the synthesis of thyroid hormone by the thyroid gland in the body. Thyroid hormones are essential for the normal development and function of the brain and nervous system, and the maintenance of body heat and energy. When people do not have enough iodine, they cannot make enough thyroid hormone. This deficiency of iodine has several important health consequences that together are called iodine deficiency disorders (IDD). Iodine deficiency frequently causes permanent brain damage and cognitive impairment in children, reproductive failure (miscarriages, stillbirths), decreased child survival, goiter, and socioeconomic stagnation. Iodine deficiency is important because of its widespread prevalence and its destructive effects on human health. Proper supplementation with iodine completely prevents these consequences. Iodine is supplemented in the form of iodized salt, iodized oil, iodized water, and frequent administration of Lugol’s iodine. Among these, salt iodization has been proven and the most effective strategy to prevent IDD at the population level [1, 2].

Iodine deficiency is also a public health important problem in Ethiopia. The national total goiter rate among Ethiopian women was above 35.8% [3]. Also, the pooled estimate of goiter among children in Ethiopia was 40.50% Thus, the government of Ethiopia recommended and implemented universal salt iodization (USI) to prevent iodine deficiency and its associated deficiency disorders [4, 5]. The availability of adequately iodized salt at the household level is one of the process indicators used to monitor the consumption of iodized salt at the population level. According to the World Health Organization recommendation, the coverage of adequately iodized salt at the household level should be above 90% to prevent iodine deficiency disorders [2].

In Ethiopia, the proportion of adequately iodized salt at the household level has been reported in several studies, which is inconsistent and ranges from 4.6% at Dega Damot Districts of Amhara region [6] to 95.5% at Kolfe Keranio sub-city of Addis Ababa [7]. As a result of variations of findings across previously existing studies, producing a pooled proportion of adequately iodized salt at the household level is needed. Therefore, this systematic review and meta-analysis were conducted to produce the pooled proportion of adequately iodized salt at the household level in Ethiopia since 2013. The pooled estimate of adequately iodized salt at the household level will be an important indicator for the government, programmers, policymakers, and different stakeholders to monitor the progress of adequately iodized salt coverage at the household level.

## Methods

### Data source and search strategy

The studies were found through internet searches using databases of PubMed, Google Scholar, and Science direct. Searching of the articles was done by AAA, DBK, MD, CTL, MAA, MYB & HTD using the keywords of “Availability”, "Adequately Iodized salt" "Household Level" "Ethiopia" in combination or individually. The last search was conducted on 10 August 2020.

### Inclusion criteria

#### Study setting

Studies conducted in Ethiopia were included.

#### Study units

Studies conducted on the availability of adequately iodized salt at the household’s level.

#### Publication status

Both published and unpublished articles were included.

#### Language

Only studies published in the English language were included.

#### Study type

Studies employed using observational study designs were included.

#### Publication year

Articles that were published between first January 2013 and 10^th^ August 2020 were included. The rationale for including those studies published since January 2013 was to generate more recent information that will be useful for decision making.

#### Type of article

Only full-text articles were included.

### Exclusion criteria

Studies that did not report the outcome of interest and studies with the unsatisfactory quality score (Newcastle Ottawa quality score ≤4) were excluded from this systematic review and meta-analysis [8].

### Screening, data extraction, and quality assessment

Before conducting data abstraction, the data extraction format was prepared in a Microsoft™ Excel spreadsheet. The data extraction sheet includes the author’s name, year of publication, study design, region, study area, residence sample size, response rate, and proportion of adequately iodized salt at the household level. Studies that fulfill the inclusion criteria were screened and extracted by AAA, DBK, MYB, CTL, MD, MAA, & YMA using the pre-defined data extraction format. Then, the two authors (AAA, DBK) done quality assessment independently for the included studies using the Newcastle-Ottawa Quality assessment scale adapted for cross-sectional studies. The quality assessment scale includes representativeness of the sample, sample size satisfactoriness, non-response rate, and validity of measurement tool, comparability of subjects in different outcome groups, outcome assessment, and statistical test [8]. The 2 reviewers each (AAA and DBK) scored the included articles based on the above-mentioned quality assessment criteria. The combined quality assessment score for each study ranges from 0–10. The two researchers who extracted the data were discussed to solve any disagreements on data extractions under the mediator of the third author (YMA). Besides, the Microsoft Word PRISMA 2009 checklist was used to compile the report [9] (S1 File).

### Outcome measurement

Adequately iodized salt at household level: If a household salt is fortified with the iodine content of ≥15 parts per million (ppm).

### Statistical analysis

The data were extracted in excel and exported into Stata version 14 for analysis. The pooled estimate was computed using the “metaprop” command [10]. The original articles were described using forest plots and tables. There was statistically significant heterogeneity among studies. Therefore, we used a random-effect model to pool the proportion of adequately iodized salt at the household level. The pooled proportion with a 95% confidence interval was reported. Sub-group analysis was done by geographic location where the study was done, residence, year of publication, and sample size. Sensitivity analysis was done to check the influence of small studies on the pooled prevalence [11].

### Heterogeneity test and publication bias

The presence of statistical heterogeneity within the included studies was evaluated using the I-squared statistic. The heterogeneity was classified as low, medium, and high when the value of I-squared was around 25%, 50%, and 75%, respectively [12]. We used Egger’s regression test to identify evidence of publication bias. Statistically significant publication bias was declared at a p-value of less than 0.05. The trim and fill analysis was done to quantify the effect sizes of missed studies [13].

## Results

### Search results

**Fig 1:**
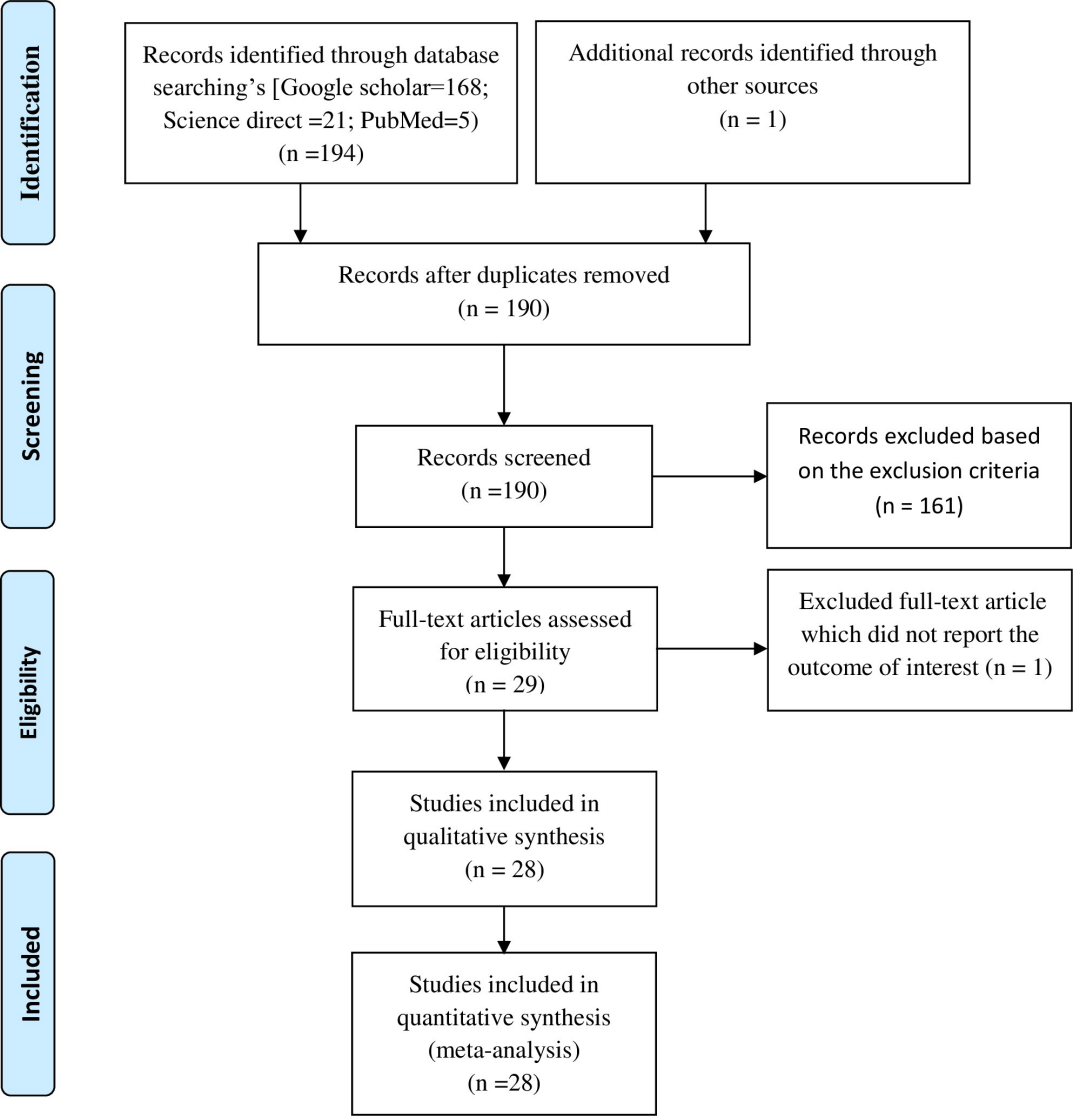
PRISMA flow chart which shows the selection of included studies.

A total of 195 studies were identified by the electronic search in PubMed, Google Scholar, and Science direct. Of which, 5 articles were excluded due to duplication, 161 were excluded based on the exclusion criteria, 1 study was excluded since they did not report the outcome of interest [14]. Finally, 28 cross-sectional studies were found to be eligible and included in the current systematic review and meta-analysis (Fig 1).

### Characteristics of reviewed studies

As shown in Table 1, a total of 28 studies (with 15561 households) met the inclusion criteria. Six regions and 2 city administrations were represented by this systematic review and meta-analysis. These are, 9 were Amhara region (n = 9) [6, 15–22], Oromia region(n = 8) [23–30], SNNPR (n = 4) [31–34], Tigray region (n = 2) [35, 36], Dire Dawa (n = 2) [37, 38], Addis Ababa (n = 2) [7, 39], and Benishangul Gumuz (n = 1) [40]. The smallest sample size (269) was reported from a study at the Sidama zone in SNNPR and the highest (1194) was from a study at the Dera district in the Amhara region. The quality score ranges from 7–10 with a quality score of good and very good. The proportion of adequately iodized salt at the household level as reported from the primary studies ranged from 4.6% at Dega Damot Districts of Amhara region [6] to 95.5% at Kolfie Keranio sub-city of Addis Ababa (7) (Table 1).

**Table 1:** Summary of the included studies which were done on the proportion of adequately iodized salt at HH level in Ethiopia, 2013–2020 (n = 28).

S.No.	Authors	Year of Publication	Region	Study Area	Study Setting	Sample size	Response Rate (%)	NOQS	Proportion HHs using AIS (%)
1	Mesele et al. [16]	2014	Amhara	Lay Armachiho	Both	694	99.4	10	29.7
2	Mekonnen et al. [19]	2018	Amhara	Dessie & Combolcha	Urban	500	95.4	10	68.8
3	Ajema et al. [31]	2020	SNNPR	Arba Minch	Urban	875	100.0	10	58.20
4	Abebe et al. [17]	2017	Amhara	Dabat	Both	705	98.7	10	33.20
5	Anteneh et al. [18]	2017	Amhara	Dera	Both	1194	96.2	10	57.2
6	Desta et al. [35]	2019	Tigray	Ahferom	Both	292	91.8	8	17.5
7	Gebriel et al. [40]	2014	Benishangul Gumuz	Assosa	Urban	395	100.0	10	26.1
8	Wondimagegn et al. [33]	2018	SNNPR	Wolaita Sodo	Both	440	99.8	10	36.7
9	Tariku et al. [20]	2019	Amhara	Mecha	Both	700	98.0	10	63.3
10	Gebremariam et al. [15]	2013	Amhara	Gondar	Urban	810	95.5	10	28.9
11	Hailu et al. [25]	2016	Oromia	Robe	Both	393	93.1	10	29.0
12	Gidey et al. [36]	2015	Tigray	Laelay Maychew	Rural	600	98.4	9	33.0
13	Yaye et al. [38]	2016	Dire Dawa	Dire Dawa	Urban	694	100	10	7.5
14	Hawas et al. [23]	2016	Oromia	Assela	Urban	513	96.4	10	62.9
15	Ayigegn et al. [7]	2020	Addis Ababa	Kolfie Keranio	Urban	541	95.5	10	95.5
16	Yazew [28]	2020	Oromia	Horro	Both	390	100	8	23.6
17	Meselech et al. [24]	2016	Oromia	Lalo Assabi	Both	768	95.0	10	8.7
18	Hiso et al. [29]	2019	Oromia	Duguda	Rural	402	100	10	30.7
19	Woyraw et al. [22]	2018	Amhara	Jabitehinan	Both	549	98.0	9	48.3
20	Aredo et al. [27]	2020	Oromia	Hetosa	Both	596	98.8	8	61.1
21	Tigabu et al. [21]	2017	Amhara	Gasgibla	Both	443	97.6	10	17.2
22	Asfaw et al. [32]	2020	SNNPR	Dewaro Zone	Both	230	NR	7	19.1
23	Fereja et al. [26]	2018	Oromia	Ada	Both	351	98.3	10	39.3
24	Afework et al. [6]	2019	Amhara	Dega Damot	Both	802	100.0	10	4.6
25	Ftwi *et al*. [37]	2018	Dire Dawa	Dire Dawa	Urban	402	99.5	10	49.0
26	Belay	2020	Addis Ababa	Kolfie Keranio	Urban	417	98.5	10	63.8
27	Stoecker et al. [34]	2020	SNNPR	Sidama Zone	Both	269	NR	7	21.0
28	Tololu et al.	2016	Oromia	Goba Town	Urban	596	99.7	9	30.0

NOQS: Newcastle Ottawa Quality Score; HH: Household; AIS: Adequately Iodized Salt.

### Pooled proportion of adequately iodized salt

**Fig 2:**
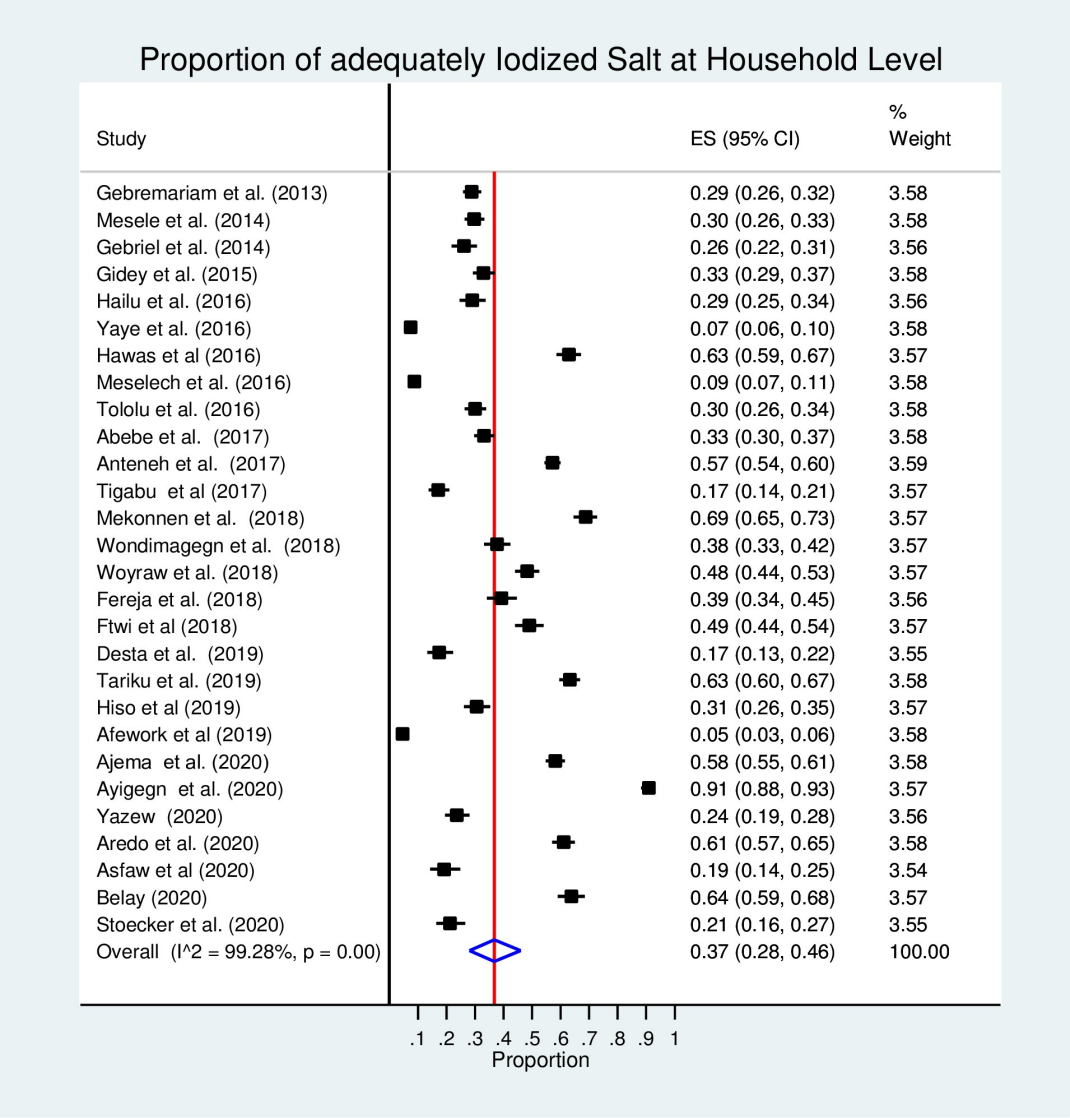
Forest plot of the 28 included studies which assessed the proportion of adequately iodized salt at HHs level in Ethiopia, 2013–2020.

The pooled proportion of adequately iodized salt at the household level was 37% (95% CI: 28, 46%; I^2^ = 99.28%, p<0.001) in Ethiopia (Fig 2).

The subgroup analyses of 28 studies by residence revealed that the pooled proportion of adequately iodized salt at a household level among rural and urban residents was 32% (95% CI: 29, 35%) and 48% (95% CI: 31, 66%), respectively. Based on 28 included studies, the subgroup analysis of adequately iodized salt proportion at the household level by regions showed that the highest proportion was found in Addis Ababa (81%; 95%CI: 78, 83%) and the lowest was found in Dire Dawa (20%; 95%CI: 17, 22%). Also, the subgroup analysis of adequately iodized salt availability at the household level was done by the year of publication. The finding revealed that the proportion of adequately iodized salt at the household level was significantly increased during 2017–2020 (42%; 95% CI: 30, 53%) as compared with 2013–2016 (27%; 95% CI: 17, 39%) (Table 2).

**Table 2:** Subgroup analysis of the pooled proportion of adequately iodized salt at the HH level in Ethiopia by region, residence, & year of publication, 2013–2020.

Variables	Subgroup	No of included Studies	Sample size	Estimated proportion of AIS at HH level % (95% CI)
**Region**	Amhara	9	6, 397	37 (23, 53)
	Oromia	8	4, 009	35 (21, 50)
	SNNPR	4	1, 814	33 (16, 53)
	Tigray	2	892	28 (25, 31)
	Addis Ababa	2	958	81 (78, 83)
	Dire Dawa	2	1, 096	20 (17, 22)
	Benishangul Gumuz	1	395	26 (22, 31)
**Residence**	Urban	10	5, 743	48 (31, 66)
	Both	16	8, 816	30 (21, 41)
	Rural	2	1, 002	32 (29,35)
**Year of Publication**	2013–2016	9	4, 867	27 (17, 39)
	2017–2020	19	10, 694	42 (30, 53)
**Total**		**38**	**15, 561**	**37 (28, 46)**

### Meta-regression

We run a random effect meta-regression by year of publication, region, residence sample size, and quality score to detect the source of heterogeneity. The finding evidenced that there is a statistically significant variation of the proportion of adequately iodized salt at HH by year of publication and residence across the pooled studies (p <0.05). The proportion of between-study variation explained by year of publication, region, residence, sample size, and quality score was 29.23% (Table 3).

**Table 3:** Meta-regression of the proportion of AIS by year of publication, region, residence, sample size, & quality score to detect the source of heterogeneity in Ethiopia, 2013–2020 (n = 28).

Variable	Coefficient	p-value	95% Conf. Interval
**Year of publication**	5.298441	0.012*	1.303038, 9.293845
**Region**	-2.753277	0.235	-7.433168, 1.926615
**Residence**	8.815902	0.039*	. .4790526, 17.15275
**Sample size**	.0108135	0.581	-.0291769, .0508039
**Quality Score**	3.172159	0.546	-7.546717, 13.89104

*Statistically significant variation.

### Publication bias and sensitivity analysis

Funnel plot and Egger regression test methods were used to check publication bias. The finding evidenced asymmetrical funnel plot and statistically significant publication bias (p<0.05). The trim and fill analysis was done to quantify the effect sizes of missed studies. The finding showed that 10 studies with negative findings were missed from publishing. Besides, the sensitivity analysis finding showed that the individual studies did not have a significant impact on the overall pooled prevalence of adequately iodized salt at the household level.

## Discussion

This systematic review and meta-analysis finding showed that the pooled estimate of adequately iodized salt at the household level in Ethiopia was 37% (95% CI: 28, 46%; I^2^ = 99.28%, p<0.001). The pooled estimate was varying by region, year of publication, and residence.

In Ethiopia, the pooled estimate of adequately iodized salt at the household level is low as compared to the world health organization’s (WHO) recommendation. According to WHO recommendation, the proportion of households with adequately iodized salt should be more than 90% to prevent iodine deficiency disorders among the population [2]. This implies that the population in Ethiopia was exposed to iodine deficiency disorders.

Based on the geographic location where the studies conducted, the highest pooled estimate was found in Addis Ababa (81%; 95%CI: 78, 83%), and the lowest prevalence found in Dire Dawa (20%; 95%CI: 17, 22). This variation might be due to weather variation across the regions, which affects the level of iodine content [34].

The subgroup analysis of adequately iodized salt by year of publication showed that the proportion of adequately iodized salt at the household level was significantly increased during 2017–2020 (42%; 95% CI: 30, 53%) as compared with 2013–2016 (27%; 95% CI: 17, 39%). This finding is in line with the finding of a study conducted based on 10 national coverage surveys in 2016 [41]. These substantial increments might be due to the government and different stakeholder’s efforts in enforcing USI laws and awareness creation on proper handling of iodized salt at the wholesaler, distributor, and household level.

The subgroup analysis of studies by residence revealed that the pooled prevalence of adequately iodized salt at the household level was higher among urban residents 48% (95% CI: 31, 66) as compared with the rural residents (32% (95% CI: 29, 35). This finding is also in line with the findings of a study conducted based on 10 national coverage surveys in 2016 [41]. Increased access to media and a high educational level in the urban area might be the possible explanations for this observed variation.

## Limitation of the study

The findings of this meta-analysis should be interpreted considering the following limitations. The first limitation is that this meta-analysis did not find a study from the two regional states of Ethiopia (Gambella and Afar) which limits the generalizability of the finding at the national level. Second, heterogeneity among the included studies was high (I^2^ statistic = 99.28%, p<0.001). Third, there is a statistically significant publication bias (p>0.05). Hence, the random effect model was used to adjust the heterogeneity among the included studies. Also, meta-regression was done to identify the source of heterogeneity. The finding evidenced that year of publication and residence were the statistically significant variables introducing such a high variation among the included studies. Furthermore, trim and fill analysis was done to treat publication bias. The analysis indicated as 10 studies were missed.

## Conclusions

In conclusion, in Ethiopia, the pooled proportion of adequately iodized salt at the household level was very low as compared to the world health organization recommendation. This indicates that the population in Ethiopia was exposed to iodine deficiency disorders. Thus, the Federal Ministry of Health of Ethiopia and different stakeholders should give more attention to improve the proportion of adequately iodized salt at the household level.

## Supporting information

S1 File PRISMA flow 2009 checklist of the study. (DOCX)

S2 File Dataset. (DTA)

## Abbreviations

AIS: Adequately Iodized Salt
CI: Confidence Interval
HH: Household
ICCIDD: International Council for Control of Iodine Deficiency Disorders
IDD: Iodine Deficiency Disorders
NOQS: Newcastle Ottawa Quality Score
PPM: Parts Per Million
SNNPR: Southern Nations Nationalities and Peoples Region
USI: Universal Salt Iodization
WHO: World Health Organization
