# Supplementary material for: Availability of adequately iodized salt at the household level in Ethiopia: A systematic review and meta-analysis
Source: PLoS One. 2021 Feb 16;16(2):e0247106. doi: 10.1371/journal.pone.0247106 (PMC7886221; doi:10.1371/journal.pone.0247106)
Supplement: Attachment — Submitted filename: Response to Reviewers.docx [file pone.0247106.s003.docx]

**Response to Reviewers**

**Response to the Academic Editor**

Dear Academic Editor,

Thank you for your invaluable comments and suggestions. We have addressed the points you raised as follow:

**1.** Please ensure that your manuscript meets PLOS ONE's style requirements, including those for file naming.

**Response:** Thank you! We have prepared the manuscript as per PLOS ONE guideline.

**2.** Please provide your rationale for restricting search timeline to 2013 onward? Why have earlier publications been excluded?

**Response:** Dear academic editor, thank you for your concern. The rationale for restricting a search timeline to 2013 onward is to generate more recent information that will be useful for decision making (Check on Method section, page 5, lines 104 & 105).

**3.** Please use standard statistical reporting of p values. For example, please delete p=0.000 and replace it with p <0.001.

**Response:** Thank you! We replaced it with p <0.001 (Check on page 10, lines 183; on page 13, line 221, and on page 14, line 250).

4. We suggest you thoroughly copyedit your manuscript for language usage, spelling, and grammar. If you do not know anyone who can help you do this, you may wish to consider employing a professional scientific editing service.

**Response:** The manuscript was thoroughly copy edited for language usage, spelling, and grammar by our colleague Mr. Fasil Wagnew who is a Lecturer at Debre Markos University (Check on “Acknowledgements Section” page 17, lines 296-298; and see the “Revised Manuscript with Track Changes”).

**Response to Reviewer #1**

Reviewer #1: This paper is, for the most part, well written, straightforward, and easy to follow. Methods are appropriate and described adequately, and the statistical tests performed seem appropriate. However, it would be beneficial to read carefully through the manuscript to make sure everything is consistent and without typos. The topic is of importance.

**Response:** Dear Reviewer, thank you for your suggestion to read the manuscript carefully in order to make sure everything is consistent and without typos. Thus, we have read and revise the manuscript (Check on the “Revised Manuscript with Track Changes”.

**Response to Reviewer #2**

**Reviewer #2:** I would recommend you to edit and reformulate the background. The use of terms such as “mental retardation” and “fertility failure” are outdated and might even be considered offensive by vulnerable populations. I would recommend you to substitute them with “cognitive impairment” and simply “infertility”, respectively. Besides these remarks, I believe the data analysis can be of great relevance for the large-scale nutritional interventions.

**Response:** Dear reviewer, thank you for your suggestion! We have edited those terms as per your suggestion (Check on page 3, line 64).

.
